# Stillbirth undercount in the sample registration system and national family health survey, India

**DOI:** 10.2471/BLT.22.288906

**Published:** 2023-01-26

**Authors:** Rakhi Dandona, Sibin George, Moutushi Majumder, Mohamed Akbar, G Anil Kumar

**Affiliations:** a Public Health Foundation of India, Plot 47, Sector 44, Gurugram, Haryana 122 002, India Gurugram, India.

## Abstract

**Objective:**

To assess the extent of under-reporting of stillbirths in India by comparing stillbirth and neonatal mortality rates from two national data sources and to review possible reasons for undercounting of stillbirths.

**Methods:**

We extracted data on stillbirth and neonatal mortality rates from the annual reports for 2016–2020 of the sample registration system, the Indian government’s main source of vital statistics. We compared the data with estimates of stillbirth and neonatal mortality rates from the fifth round of the Indian national family health survey covering events from 2016–2021. We reviewed the questionnaires and manuals from both surveys and compared the sample registration system’s verbal autopsy tool with other international tools.

**Findings:**

The stillbirth rate for India from the national family health survey (9.7 stillbirths per 1000 births; 95% confidence interval: 9.2–10.1) was 2.6 times higher than the average rate reported in the sample registration system over 2016–2020 (3.8 stillbirths per 1000 births). However, neonatal mortality rates in the two data sources were similar. We identified issues with the definition of stillbirth, documentation of gestation period, and categorization of miscarriages and abortions that could result in undercounting stillbirths in the sample registration system. In the national family health survey only one adverse pregnancy outcome is documented, irrespective of the number of adverse pregnancy outcomes in the given period.

**Conclusion:**

For India to attain its 2030 target of single-digit stillbirth rate and to monitor actions to end preventable stillbirths, efforts are needed to improve the documentation of stillbirths in its data collection systems.

## Résumé

**Objectif:**

Déterminer l'étendue de la sous-déclaration des mortinaissances en Inde en comparant les taux de mortinatalité et de mortalité néonatale provenant de deux sources de données nationales, et examiner les raisons susceptibles d'expliquer ce sous-dénombrement.

**Méthodes:**

Nous avons extrait les informations relatives aux taux de mortinatalité et de mortalité néonatale des rapports annuels, pour 2016–2020, du système de recueil de données sur des échantillons de population, qui constitue la principale source de statistiques démographiques pour le gouvernement indien. Nous avons ensuite comparé ces informations avec les taux de mortinatalité et de mortalité néonatale estimés lors de la cinquième édition de l'enquête nationale sur la santé familiale, qui couvre les événements survenus entre 2016 et 2021. Enfin, nous avons étudié les questionnaires et manuels des deux enquêtes et confronté l'outil d'autopsie verbale du système de recueil de données sur des échantillons de population à d'autres outils internationaux.

**Résultats:**

Le taux de mortinatalité figurant dans l'enquête nationale sur la santé familiale en Inde (9,7 mortinaissances pour 1000 naissances; intervalle de confiance de 95%: 9,2–10,1) était 2,6 fois plus élevé que le taux moyen mentionné dans le système de recueil de données sur des échantillons de population pour la période comprise entre 2016 et 2020 (3,8 mortinaissances pour 1000 naissances). En revanche, les taux de mortalité néonatale des deux sources d'information se sont révélés similaires. Nous avons identifié des problèmes dans la définition de la mortinatalité, la documentation relative à la période de gestation et la classification des fausses couches et avortements, ce qui pourrait entraîner un sous-dénombrement des mortinaissances dans le système de recueil de données sur des échantillons de population. L'enquête nationale sur la santé familiale n'évoque qu'une seule issue défavorable de la grossesse, sans tenir compte du nombre d'issues défavorables observées durant cette période.

**Conclusion:**

Des efforts sont nécessaires pour améliorer la documentation sur la mortinatalité dans les systèmes de recueil de données si l'Inde veut atteindre son objectif d'ici 2030: afficher un taux de mortinatalité à un chiffre et assurer un suivi des mesures visant à éliminer les mortinaissances évitables.

## Resumen

**Objetivo:**

Evaluar el grado de falta de notificación de los mortinatos en la India mediante la comparación de las tasas de mortinatalidad y mortalidad neonatal de dos fuentes de datos nacionales y revisar las posibles razones del subconteo de los mortinatos.

**Métodos:**

Se extrajeron datos sobre las tasas de mortinatalidad y mortalidad neonatal a partir de los informes anuales de 2016 a 2020 del sistema de registro de muestreo, la principal fuente de estadísticas vitales del gobierno indio. Se compararon los datos con las estimaciones de las tasas de mortinatalidad y mortalidad neonatal de la quinta ronda de la encuesta nacional de salud familiar de la India, que abarca los eventos de 2016 a 2021. Asimismo, se revisaron los cuestionarios y manuales de ambas encuestas y se comparó la herramienta de autopsia verbal del sistema de registro de muestreo con otras herramientas internacionales.

**Resultados:**

La tasa de mortinatalidad registrada en la India a partir de la encuesta nacional de salud familiar (9,7 mortinatos por cada 1000 nacimientos; intervalo de confianza del 95 %: 9,2-10,1) fue 2,6 veces superior a la tasa media notificada en el sistema de registro de muestreo entre 2016 y 2020 (3,8 mortinatos por cada 1000 nacimientos). Sin embargo, las tasas de mortalidad neonatal en las dos fuentes de datos fueron similares. Se identificaron problemas con la definición de mortinato, la documentación del periodo de gestación y la categorización de abortos espontáneos y abortos provocados que podrían dar lugar a un subconteo de mortinatos en el sistema de registro de muestreo. En la encuesta nacional de salud familiar solo se documenta un desenlace adverso del embarazo, sin considerar la cantidad de desenlaces adversos del embarazo en el periodo dado.

**Conclusión:**

La India debe esforzarse por mejorar la documentación de los mortinatos en sus sistemas de recopilación de datos a fin de alcanzar su objetivo para 2030 de lograr una tasa de mortinatalidad de un solo dígito y controlar las medidas para reducir los mortinatos evitables.

## ملخص

**الغرض:**

تقييم مدى الإبلاغ المنخفض عن حالات وفيات الرُضّع في الهند عن طريق مقارنة معدلات وفيات الرُضّع ووفيات حديثي الولادة من مصدري البيانات الوطنية، ومراجعة الأسباب المحتملة لتسجيل عدد منخفض لوفيات الرُضّع.

**الطريقة:**

قمنا باستخراج البيانات عن معدلات وفيات الرُضّع ووفيات حديثي الولادة من التقارير السنوية للفترة 2016 إلى 2020 لنظام تسجيل العينات، وهي المصدر الرئيسي للإحصاءات الحيوية لدى الحكومة الهندية. قمنا بمقارنة البيانات مع تقديرات وفيات الرُضّع ومعدلات حديثي الوفيات، المأخوذة من الجولة الخامسة للمسح الوطني الهندي لصحة الأسرة الذي يغطي الأحداث من 2016 إلى 2021. قمنا بمراجعة الاستبيانات والأدلة من كلا المسحين، وقارننا أداة تشريح الجثة السردي لنظام تسجيل العينات، مع الأدوات الدولية الأخرى.

**النتائج:**

كان معدل وفيات الرُضّع في الهند من المسح الوطني لصحة الأسرة (9.7 حالات وفيات رُضّع لكل 1000 حالة ولادة؛ بفاصل ثقة مقداره %95: 9.2 إلى 10.1) كان أعلى بمقدار 2.6 مرة عن المعدل المتوسط المبلغ عنه في نظام تسجيل العينة خلال الفترة من 2016 إلى 2020 (3.8 حالات وفيات رُضّع لكل 1000 حالة ولادة). ومع ذلك، فإن معدلات وفيات حديثي الولادة في مصدري البيانات، كانت متشابهة. قمنا بتحديد المشكلات المتعلقة بتعريف وفيات الرُضّع، وتوثيق فترة الحمل، وتصنيف حالات الإجهاض وفقدان الحمل، التي يمكن أن تؤدي إلى تقليل عدد وفيات الرُضّع في نظام تسجيل العينة. في المسح الوطني لصحة الأسرة، تم توثيق نتيجة حمل سلبية واحدة فقط، بغض النظر عن عدد نتائج الحمل السلبية في الفترة المحددة.

**الاستنتاج:**

لكي تحقق الهند هدفها لعام 2030، والمتمثل في معدل وفيات الرُضّع أحادي الرقم، ولمراقبة الإجراءات لإنهاء حالات وفيات الرُضّع التي يمكن الوقاية منها، فإن هناك حاجة لبذل جهود لتحسين توثيق حالات وفيات الرُضّع في أنظمة جمع البيانات الخاصة بها.

## 摘要

**目的:**

通过比较来自两个国家数据源的死产率和新生儿死亡率，评估印度死产漏报的程度，并审查可能导致死产漏报的原因。

**方法:**

我们从样本登记系统的 2016-2020 年年度报告中提取了死产和新生儿死亡率的数据，样本登记系统是印度政府生命统计数据的主要来源。我们将这些数据与印度第五次全国家庭健康调查（涵盖 2016-2021 年事件）中的死产和新生儿死亡率的估计值进行了比较。我们审查了两项调查的问卷和手册，并将样本登记系统的死因推断工具与其他国际工具进行了比较。

**结果:**

印度全国家庭健康调查中的死产率（每 1000 例分娩中有 9.7 例死产；95% 置信区间：9.2-10.1）比 2016-2020 年期间样本登记系统报告的平均死产率（每 1000 例分娩中有 3.8 例死产）高出 2.6 倍。然而，两个数据源的新生儿死亡率相似。我们发现在死产的定义、妊娠期的记录以及流产和堕胎的分类方面存在问题，这些问题可能导致样本登记系统中的死产漏报。在全国家庭健康调查中，只记录了一种不良妊娠结果，而未考虑指定时期不良妊娠结果的数量。

**结论:**

印度需要努力改进其数据收集系统中的死产记录，才能实现其 2030 年个位数死产率的目标，并监督实施措施以避免可预防死产的发生。

## Резюме

**Цель:**

Оценить степень занижения данных о мертворождениях в Индии путем сравнения показателей мертворождений и смертности новорожденных, полученных из двух национальных источников данных, и рассмотреть возможные причины занижения статистических показателей мертворождений.

**Методы:**

Извлечены данные о показателях мертворождений и смертности новорожденных из ежегодных отчетов за 2016–2020 гг. системы выборочной регистрации – основного источника статистики естественного движения населения для Правительства Индии. Полученные данные сравнивались с оценками показателей мертворождений и смертности новорожденных, полученными в ходе пятого раунда национального исследования состояния здоровья семей в Индии, охватывающего события 2016–2021 гг. Во время проведения обоих исследований были изучены опросники и руководства, а также проведено сравнение инструмента вербальной аутопсии в системе выборочной регистрации с другими международными инструментами.

**Результаты:**

Коэффициент мертворождений в Индии по результатам национального исследования состояния здоровья семей (9,7 мертворождения на 1000 родов; 95%-й ДИ: 9,2–10,1) в 2,6 раза превышал средний коэффициент, указанный в системе выборочной регистрации на 2016–2020 гг. (3,8 мертворождения на 1000 родов). Однако показатели смертности новорожденных в двух источниках данных были схожи. Выявлены проблемы с определением случаев мертворождения, документированием гестационного срока беременности и распределением выкидышей и абортов по категориям, которые могут привести к занижению статистических показателей мертворождений в системе выборочной регистрации. В национальном исследовании состояния здоровья семей задокументирован только один неблагоприятный исход беременности, независимо от количества неблагоприятных исходов беременности за указанный период.

**Вывод:**

Для того чтобы Индия достигла поставленной на 2030 год цели по достижению однозначного показателя мертворождений и мониторинга действий по устранению предотвратимых случаев мертворождения, необходимы действия по оптимизации процессов документирования случаев мертворождения в системах сбора данных.

## Introduction

A late-gestation fetal death occurring at or after 28 weeks of gestation is defined as a stillbirth.^1^ In 2021, a systematic review found a 28% increase in stillbirths during the coronavirus disease-2019 (COVID-19) pandemic,^2^ possibly as a result of restrictions in the availability and use of maternal health services.^3^ Despite the overlap between the causes of and effective interventions for prevention of stillbirths and neonatal deaths,^4^^,^^5^ stillbirths have been neglected in the global policy agenda. Inclusion of stillbirths in the Every Newborn Action Plan of 2020,^6^^,^^7^ and the stillbirth estimates generated by the United Nations Inter-Agency Group for Child Mortality Estimation, signalled increased attention on stillbirth prevention.^8^

The burden of stillbirths in India is high. According to the Inter-Agency Group report, an estimated 340 600 stillbirths occurred in the Indian population of 1.4 billion in 2019, the largest numbers globally, translating into a rate of 13.9 stillbirths per 1000 births (95% confidence interval, CI: 11.4–17.0).^8^ However, this is a modelled estimate because stillbirths are believed to be under-reported in India’s main source of vital statistics: the Sample Registration System.^9^^,^^10^ The estimated stillbirth rate from this source for India was 3 stillbirths per 1000 births in 2020, which is 4.6 times lower than the Inter-Agency Group estimate.^8^^,^^11^ Another data source for stillbirths for India and its states is the National Family Health Survey, the equivalent of the demographic and health surveys that are done in many countries.^12^ However, the national family health survey was not used in the Inter-Agency Group modelled estimate as the data did not meet the inclusion criteria.^8^

Using the modelled estimates, the Indian government launched the India Newborn Action Plan with a commitment to achieving a stillbirth rate below 10 stillbirths per 1000 births by 2030, with all the states to achieve this target individually by 2035.^9^ The action plan aims to realize the health ministry’s goal of ending preventable stillbirths and neonatal deaths with specific targets and interventions. Importantly, the action plan recognizes that accurate measuring of stillbirth rates is crucial to improve both maternal and newborn survival.^13^ In view of the high burden of stillbirths, the underestimation of stillbirths and the possibility of an increase in stillbirths due to the COVID-19 pandemic, it is important for India to improve the robustness of reporting the stillbirth rate in its main data source to monitor progress towards the action plan 2030 goal. 

With this background, we undertook a comparative assessment of stillbirths reported in India’s sample registration system and national family health survey to determine the extent of under-reporting in stillbirths in the former system. We also compared the neonatal mortality rates between these data sources to highlight issues that may be specific to stillbirths. To understand possible reasons for undercounting of stillbirths, we reviewed the questionnaires and manuals from the two data sources to determine how data on stillbirths are collected. 

## Methods

### Data sources

**Fig. 1:**
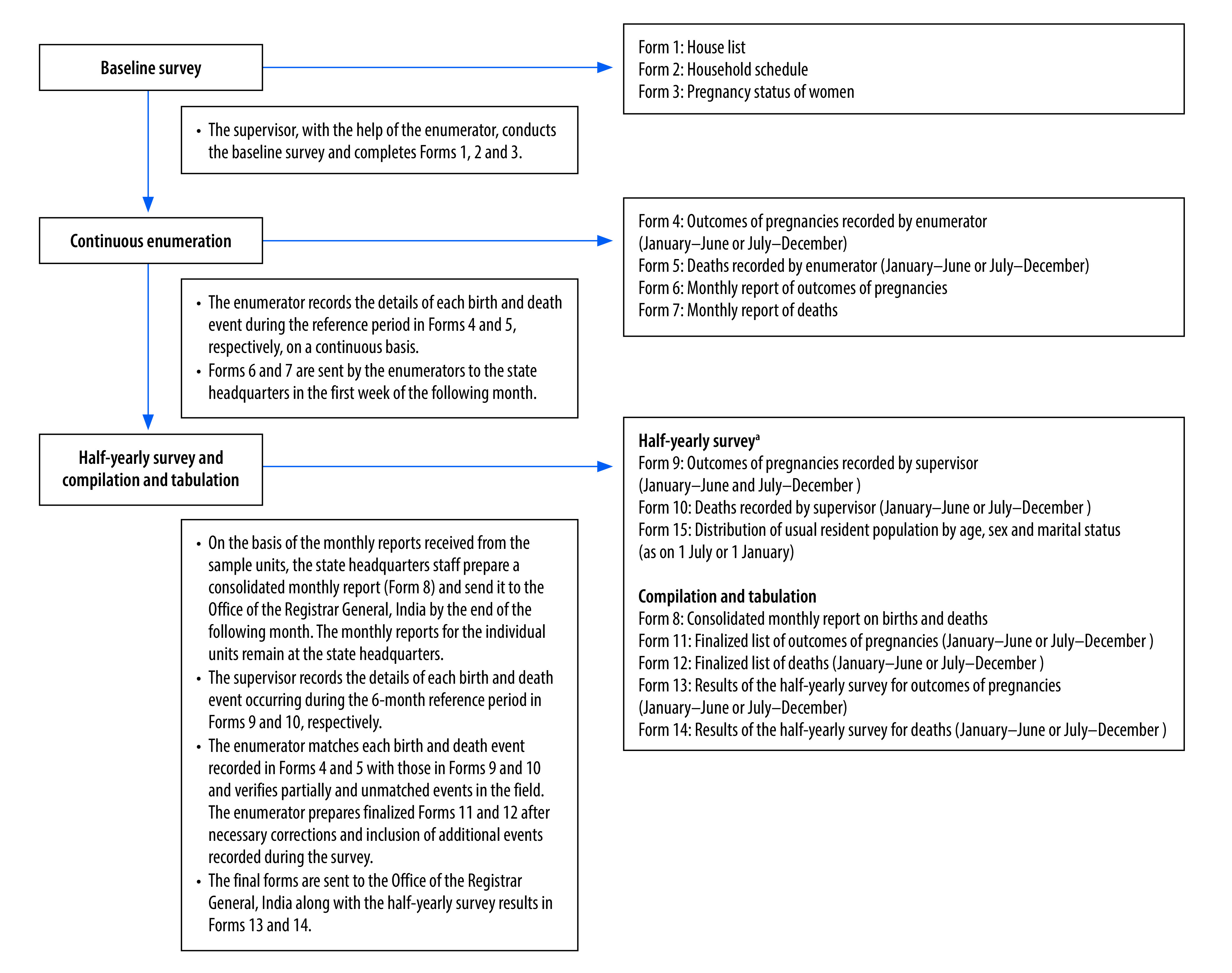
Process and forms used for data collection in the sample registration system, India

^a^ Only the forms relevant to the birth and death registration in sample registration system are shown.

The sample registration system is a large, routine demographic survey in India with the primary objective to provide reliable estimates of birth, death and infant mortality rates at the state level. The rolling survey reports annually and is conducted under the oversight of the Registrar General of India.^14^ The sample frame for the registration system covered 8861 primary sampling units randomly selected from the preceding census to be representative of the population at the state level. The flowchart of data collection is shown in Fig. 1.^15^^,^^16^ The selected households are continuously monitored for vital events by two independent surveyors. One part-time enumerator visits the home every month, and a full-time surveyor visits the home every 6 months. Both independently record the births and deaths in the household for a 6-month period. A third staff member compares the two reports, arriving at a final list of births and deaths for each household, which completes each half-yearly survey. The surveyors each cover about 150 households with a total average population of 900, and report about 50 deaths and 150 births every 6 months. Verbal autopsy is used to estimate cause-specific mortality. The supervisor conducts the verbal autopsy interview with the close relative of the deceased. There is an extensive system of matching and re-matching of vital events, and monitoring to maintain the quality of data collected.^15^

The national family health survey is planned under the oversight of India’s Ministry of Health and Family Welfare with support from ORC Macro, United States of America, and other agencies.^12^ The primary objective of the survey is to provide data on reproductive health and family planning, along with prevalence estimates of various diseases or conditions. We used primary data from the fifth round of the survey conducted in 2019–2021, documenting births in the previous 5 years across the Indian states for all states in India.^17^ The survey had 30 456 primary sampling units, selected from across the country, with the 2011 census serving as the sampling frame. For our analysis, we focused on the women’s questionnaire used in the survey.^17^ Ever-married women aged 15–49 years responded to questions on a large variety of topics, including full history of live births and the last adverse pregnancy outcome in the previous 5 years (documented from 2014 onwards), irrespective of the number of pregnancies. The fieldwork was conducted by 17 field agencies with interviewers trained in the survey procedures.

No ethical approval was required for this analysis because we used reports, manuals and questionnaires available in the public domain.

### Data analysis

#### Mortality rates

For the sample registration system, we were able to extract data directly from the annual reports available from 2016 to 2020.^18^ We then calculated the average stillbirth and neonatal mortality rates for the five-year period 2016–2020.

For the national family health survey, we needed to compute the stillbirth rate from the women’s data set in the survey. We did this in two different ways: (i) by direct estimation of the number of stillbirths, from responses to the question: “Did that pregnancy [the last one] end in a miscarriage, an abortion, or a stillbirth?” and (ii) after adjusting the stillbirth data based on the gestation period, from responses to the question: “How many months pregnant were you when the last such pregnancy ended?”^17^ For the adjustment, we considered the miscarriages and abortions reported with a gestation period of 7 months or longer also as stillbirths. Stillbirths reported with a gestation period shorter than 7 months were not considered as stillbirths (more details are in the authors’ online repository).^19^ Stillbirth rate was the number of stillbirths divided by the number of births. We computed the neonatal mortality rate for India and each state from the survey birth data set using the denominator for live births from 2016 to 2021, from responses to the question: “On what day, month and year was [NAME] born?” and neonatal deaths with age at death between 0 to 27 days of birth as the numerator for these live births, from responses to the question: “If dead, how old was [NAME] when he/she died?”

We compared the average stillbirth and neonatal mortality rates from the sample registration for years 2016–2020 and with those obtained in the fifth national family health survey for years 2019–2021 at the country- and state-level. As only 1641 (0.82%) of the 196 080 births in the national family health survey were in 2021, we included the year 2021 births for this analysis. The national family health survey provides data for all 30 states of India, whereas sample registration provides stillbirth and neonatal mortality rates only for 22 large states. We used Stata version 13 (Stata Corp., College Station, USA) and Excel (Microsoft Corp., Redmond, USA) for this analysis, and report statistical significance by rate ratio test where applicable.

#### Stillbirth documentation

**Fig. 2:**
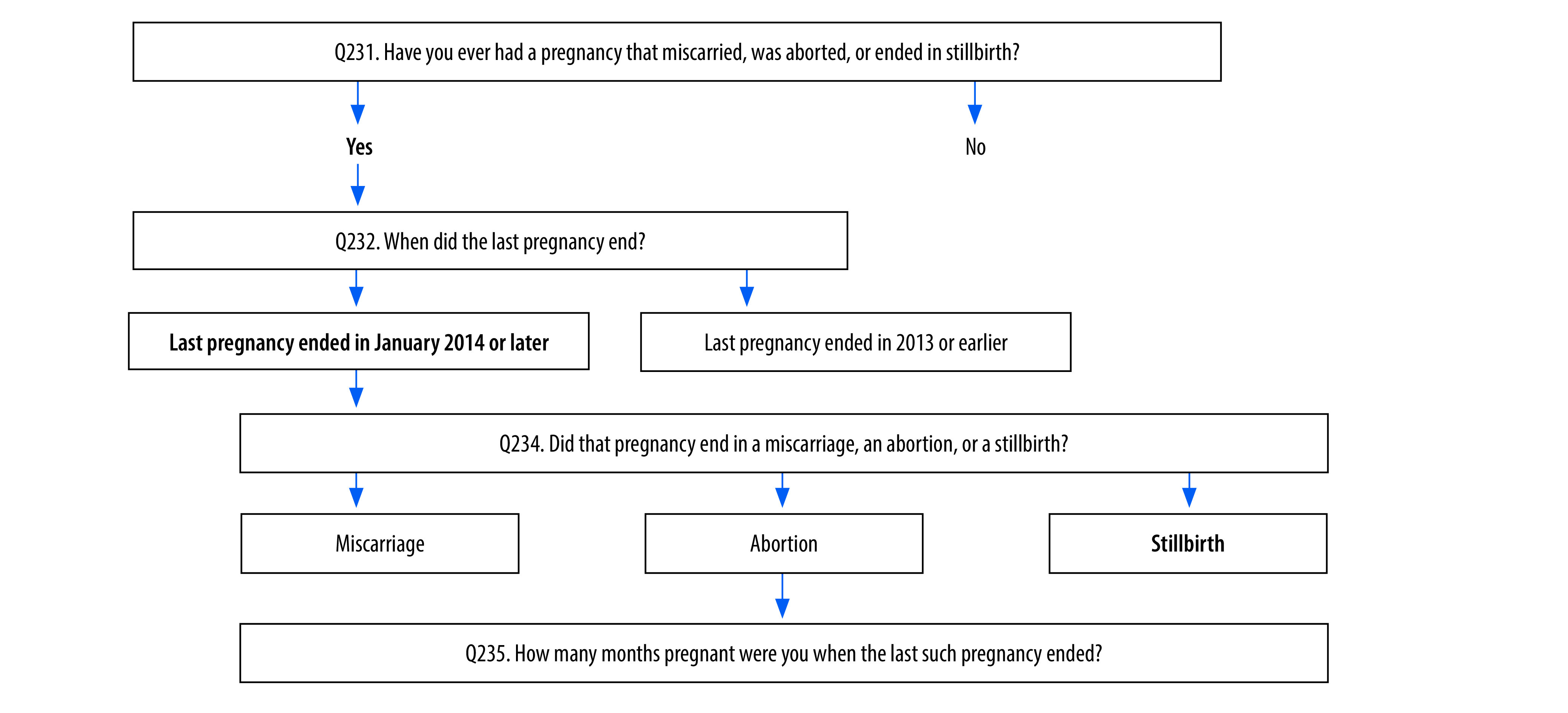
Documentation of stillbirths in the women’s questionnaire in the fifth round of the national family health survey, India

Note: Bold font indicates the data documentation process to arrive at the stillbirth estimates.

To understand possible reasons for the differences in stillbirth rates, we reviewed the questions used to collect data on stillbirths in both surveys. The national family health survey women’s questionnaire was readily available in the public domain, and the relevant questions are shown in Fig. 2.^17^ Despite several attempts, we were unable to locate the sample registration system forms listed in Fig. 1. However, the enumerator manual available in the public domain^16^ provided enough detail to understand how births were recorded. 

The sample registration system used a verbal autopsy interview to document the cause of death. This tool also has questions to differentiate between a neonatal death soon after birth and a stillbirth^20^ (more information is in the online repository).^19^ Verbal autopsy is a method for estimating population-level causes of death in populations without a complete vital registration system. The information is obtained from the dead infant’s caretaker, by trained interviewers through verbal autopsy interviews. These interviews include the circumstances, signs and symptoms of a variety of diseases and conditions to determine the likely cause of death, the health care that was sought in the period leading up to the infant’s death, and the circumstances of events leading to the death, as narrated by the respondent.^21^^,^^22^ The cause of death can be determined from the verbal autopsy by physicians’ review or automated computer algorithms. We compared the process questions used to identify stillbirths in the sample registration system’s verbal autopsy tool with those in two other verbal autopsy tools routinely used in low- and middle- income country settings: the Population Health Metrics Research Consortium verbal autopsy tool^23^ and the World Health Organization (WHO) verbal autopsy tool^24^ (summarized in the online repository).^19^

We also reviewed the training manuals for national family health survey interviewers and for sample registration system enumerators, supervisors and physicians to understand how they are trained to collect data on stillbirths.^15^^,^^25^^,^^26^

## Results

### Stillbirths

The annual stillbirth rate reported in the sample registration system ranged from 5.0 stillbirths per 1000 births in 2016 to 3.0 stillbirths per 1000 births in 2019 and 2020, with an average of 3.8 stillbirths per 1000 births over the 5-year period (Table 1). For most states, the stillbirth rate was below 10 stillbirths per 1000 births, with some exceptions in 2016, 2017 and 2020. Stillbirths continued at a steady rate or fell between 2016 and 2020 for most states, except six states. One state had a stillbirth rate of 10 or more stillbirths per 1000 births in 2020.^19^

**Table 1:** Stillbirth and neonatal mortality rates from the sample registration system, India, 2016–2020

State	Stillbirths per 1000 births						Neonatal deaths per 1000 live births				
	2016	2017	2018	2019	2020		2016	2017	2018	2019	2020
All States	4.0	5.0	4.0	3.0	3.0		24.0	23.0	23.0	22.0	20.0
Andhra Pradesh	3.0	3.0	3.0	1.0	1.0		23.0	23.0	21.0	18.0	17.0
Arunachal Pradesh	NA	NA	NA	NA	NA		NA	NA	NA	NA	NA
Assam	2.0	2.0	2.0	2.0	3.0		23.0	22.0	21.0	20.0	19.0
Bihar	3.0	2.0	2.0	1.0	1.0		27.0	28.0	25.0	23.0	21.0
Chhattisgarh	10.0	13.0	9.0	9.0	6.0		26.0	26.0	29.0	28.0	26.0
Delhi	4.0	5.0	5.0	1.0	0		12.0	14.0	10.0	8.0	9.0
Goa	NA	NA	NA	NA	NA		NA	NA	NA	NA	NA
Gujarat	6.0	5.0	4.0	3.0	4.0		21.0	21.0	19.0	17.0	16.0
Haryana	5.0	9.0	6.0	5.0	7.0		22.0	21.0	22.0	19.0	19.0
Himachal Pradesh	24.0	13.0	7.0	5.0	4.0		16.0	14.0	13.0	13.0	13.0
Jammu and Kashmir^a^	2.0	1.0	1.0	1.0	3.0		18.0	17.0	17.0	15.0	12.0
Jharkhand	NA	1.0	1.0	1.0	2.0		21.0	20.0	21.0	19.0	17.0
Karnataka	6.0	6.0	5.0	5.0	3.0		18.0	18.0	16.0	16.0	14.0
Kerala	6.0	7.0	5.0	3.0	4.0		6.0	5.0	5.0	5.0	4.0
Madhya Pradesh	8.0	6.0	5.0	6.0	5.0		32.0	33.0	35.0	33.0	31.0
Maharashtra	4.0	5.0	5.0	3.0	3.0		13.0	13.0	13.0	13.0	11.0
Manipur	NA	NA	NA	NA	NA		NA	NA	NA	NA	NA
Meghalaya	NA	NA	NA	NA	NA		NA	NA	NA	NA	NA
Mizoram	NA	NA	NA	NA	NA		NA	NA	NA	NA	NA
Nagaland	NA	NA	NA	NA	NA		NA	NA	NA	NA	NA
Odisha	13.0	12.0	10.0	8.0	10.0		32.0	32.0	31.0	30.0	28.0
Punjab	6.0	5.0	5.0	3.0	3.0		13.0	13.0	13.0	12.0	12.0
Rajasthan	3.0	8.0	6.0	3.0	4.0		28.0	27.0	26.0	25.0	23.0
Sikkim	NA	NA	NA	NA	NA		NA	NA	NA	NA	NA
Tamil Nadu	3.0	3.0	4.0	4.0	2.0		12.0	11.0	10.0	10.0	9.0
Telangana	1.0	1.0	2.0	0.0	2.0		21.0	20.0	19.0	17.0	15.0
Tripura	NA	NA	NA	NA	NA		NA	NA	NA	NA	NA
Uttar Pradesh	3.0	3.0	3.0	2.0	4.0		30.0	30.0	32.0	30.0	28.0
Uttarakhand	9.0	11.0	8.0	3.0	6.0		30.0	24.0	22.0	19.0	17.0
West Bengal	3.0	5.0	5.0	5.0	4.0		17.0	17.0	16.0	15.0	14.0

NA: not available.

^a^ Ladakh is included with Jammu and Kashmir.

Source: We obtained the sample registration system data from the reports available from the Office of the Registrar General & Census Commissioner India.^27^ The numbers of births, stillbirths and neonatal deaths were not reported in the sample registration system reports.

Based on the estimates from the women’s data set in the national family health survey, the total number of stillbirths reported over the study period 2016–2021 was 1914,^19^ giving an overall rate of 9.7 stillbirths per 1000 births (95% CI: 9.2–10.1; Table 2; maps are available in the online repository).^19^ After adjusting for gestation period,^19^ the stillbirth numbers were revised up to 2225, giving an estimated rate of 11.2 stillbirths per 1000 births (95% CI: 10.8–11.7), an increase of 15% compared with the direct estimate. In five of the states (Andhra Pradesh, Kerala, Maharashtra, Mizoram and Sikkim) the adjusted stillbirth rate increased by 50% or more, but the change was not statistically significant in any of these states.^19^

**Table 2:** Stillbirth rates from the fifth national family health survey 2016–2021, and average stillbirth rates from the sample registration system over 2016–2020, India

State	National family health survey				Sample registration system		Difference in stillbirth rates	
	Stillbirth rate per 1000 births (95% CI)		% change in stillbirth rate after adjustment		Average annual stillbirth rate per 1000 births^b^		Ratio of national family health survey direct estimate to sample registration system value	Ratio of national family health survey adjusted estimate to sample registration system value
	Direct estimate^a^	Adjusted estimate^a^						
All States	9.7 (9.2–10.1)	11.2 (10.8–11.7)	15		3.8		2.6	2.9
Andhra Pradesh	5.9 (3.3–10.3)	11.2 (7.4–16.8)	90		2.2		2.7	5.1
Arunachal Pradesh	5.5 (3.9–8.0)	5.9 (4.2–8.4)	7		NA		NA	NA
Assam	9.2 (7.3–11.6)	10.1 (8.1–12.6)	10		2.2		4.2	4.6
Bihar	13.0 (11.4–14.9)	13.9 (12.2–15.9)	7		1.8		7.2	7.7
Chhattisgarh	10.8 (8.8–13.3)	14.8 (12.4–17.6)	37		9.4		1.1	1.6
Delhi	9.4 (6.4–13.9)	12.4 (8.8–17.4)	32		3.0		3.1	4.1
Goa	0.0	0.0	NA		NA		NA	NA
Gujarat	6.3 (4.7–8.4)	7.0 (5.3–9.2)	11		4.4		1.4	1.6
Haryana	11.6 (9.3–14.5)	13.5 (11.0–16.5)	16		6.4		1.8	2.1
Himachal Pradesh	3.2 (1.4–7.0)	4.7 (2.5–9.1)	47		10.6		0.3	0.4
Jammu and Kashmir^c^	10.7 (8.1–14.0)	9.9 (7.4–13.1)	−7		1.6		6.7	6.2
Jharkhand	12.5 (10.4–14.9)	15.3 (13.1–17.9)	22		1.3		9.6	11.8
Karnataka	6.3 (4.6–8.6)	7.9 (6.0–10.4)	25		5.0		1.3	1.6
Kerala	1.5 (0.5–4.7)	3.5 (1.7–7.4)	133		5.0		0.3	0.7
Madhya Pradesh	8.5 (7.2–10.1)	10.7 (9.2–12.4)	26		6.0		1.4	1.8
Maharashtra	6.3 (4.7–8.5)	9.8 (7.7–12.4)	56		4.0		1.6	2.5
Manipur	7.3 (4.5–11.6)	9.8 (6.5–14.7)	34		NA		NA	NA
Meghalaya	10.5 (8.0–13.8)	8.9 (6.6–11.9)	−15		NA		NA	NA
Mizoram	1.7 (0.6–5.3)	4.6 (2.3–9.2)	171		NA		NA	NA
Nagaland	4.9 (2.7–8.9)	5.8 (3.4–10.0)	18		NA		NA	NA
Odisha	13.7 (11.4–16.4)	15.3 (12.9–18.2)	12		10.6		1.3	1.4
Punjab	6.7 (4.8–9.3)	8.8 (6.6–11.7)	31		4.4		1.5	2.0
Rajasthan	8.3 (6.9–10.1)	7.7 (6.3–9.4)	−7		4.8		1.7	1.6
Sikkim	2.1 (0.3–14.8)	10.4 (4.3–24.9)	395		NA		NA	NA
Tamil Nadu	5.8 (4.2–8.0)	6.8 (5.0–9.1)	17		3.2		1.8	2.1
Telangana	7.8 (5.7–10.5)	9.4 (7.2–12.4)	21		1.2		6.5	7.8
Tripura	13.9 (9.1–21.3)	13.9 (9.1–21.3)	0		NA		NA	NA
Uttar Pradesh	12.4 (11.3–13.7)	13.9 (12.7–15.2)	12		3.0		4.1	4.6
Uttarakhand	12.6 (9.4–16.8)	14.5 (11.1–19.0)	15		7.4		1.7	2.0
West Bengal	9.7 (7.1–13.1)	14.4 (11.2–18.5)	48		4.4		2.2	3.3

CI: confidence interval; NA: not available.

^a^ We estimated the stillbirth rate from responses to questions in the women’s data set of the national family health survey for the years 2016–2021. We report the direct estimates of stillbirths and the estimates when stillbirths were adjusted based on the gestation period.

^b^ We calculated the 5-year average stillbirth rate from the rates reported in the annual sample registration system reports, 2016–2020.

^c^ Ladakh is included with Jammu and Kashmir.

Sources: We obtained the sample registration system data from the reports available from the Office of the Registrar General & Census Commissioner India^27^ and the national family health survey data from the Demographic and Health Surveys website.^28^ The sample registration system does not provide confidence interval around the point estimates. The numbers of births, stillbirths and neonatal deaths were not reported in the sample registration system reports.

The overall direct estimate of the stillbirth rate from the national family health survey was 2.6 times higher than the average rate from the sample registration system. This ratio ranged from 0.3 to 9.6 at the state level (Table 2). Using the adjusted stillbirth rates from the women’s data set, the overall stillbirth rate in the national family health survey was 2.9 times higher than the sample registration stillbirth rate, with this ratio ranging from 0.4 to 12.2 across the states.

### Neonatal deaths

The overall neonatal mortality rate from the sample registration system ranged from 24.0 neonatal deaths per 1000 in 2016 to 20.0 per 1000 live births in 2020 (Table 1), with an average of 22.4 neonatal deaths per 1000 live births during this period. The neonatal mortality rate from the national family health survey was similar, at 24.9 neonatal deaths per 1000 live births (95% CI: 24.3–25.6), a ratio of 1.1 when comparing the estimates of the two surveys. These ratios ranged from 0.5 to 1.8 across the states, with eight states having a ratio between 0.9 and 1.1 (Table 3).

**Table 3:** Neonatal mortality rate from the fifth national family health survey 2016–2021 and average neonatal mortality rate from the sample registration system over 2016–2020, India

State	Neonatal death rate per 1000 live births		Ratio of national family health survey value to sample registration system value
	National family health survey (95% CI)	Sample registration system^a^	
All States	24.9 (24.3–25.6)	22.4	1.1
Andhra Pradesh	20.7 (15.3–27.9)	20.4	1.0
Arunachal Pradesh	8.3 (6.1–11.1)	NA	NA
Assam	23.2 (20.1–26.8)	21.0	1.1
Bihar	34.3 (31.5–37.2)	24.8	1.4
Chhattisgarh	30.2 (26.6–34.1)	27.0	1.1
Delhi	16.0 (11.9–21.6)	10.6	1.5
Goa	3.8 (0.5–26.5)	NA	NA
Gujarat	21.8 (18.6–25.4)	18.8	1.2
Haryana	21.0 (17.8–24.7)	20.6	1.0
Himachal Pradesh	20.6 (15.1–28.0)	13.8	1.5
Jammu and Kashmir^b^	9.3 (7.0–12.5)	15.8	0.6
Jharkhand	28.2 (25.0–31.7)	19.6	1.4
Karnataka	16.4 (13.5–19.9)	16.4	1.0
Kerala	2.5 (1.1–6.1)	5.0	0.5
Madhya Pradesh	29.6 (27.0–32.4)	32.8	0.9
Maharashtra	20.1 (17.0–23.7)	12.6	1.6
Manipur	17.2 (12.6–23.4)	NA	NA
Meghalaya	20.2 (16.6–24.6)	NA	NA
Mizoram	8.7 (5.2–14.3)	NA	NA
Nagaland	12.2 (8.4–17.7)	NA	NA
Odisha	30.5 (27.0–34.5)	30.6	1.0
Punjab	22.9 (19.2–27.3)	12.6	1.8
Rajasthan	20.5 (18.2–23.1)	25.8	0.8
Sikkim	8.4 (3.2–22.3)	NA	NA
Tamil Nadu	12.8 (10.3–15.9)	10.4	1.2
Telangana	19.7 (16.3–23.8)	18.4	1.1
Tripura	26.3 (19.2–35.7)	NA	NA
Uttar Pradesh	36.2 (34.3–38.3)	30.0	1.2
Uttarakhand	28.1 (23.1–34.1)	22.4	1.3
West Bengal	18.3 (14.6–22.9)	15.8	1.2

CI: confidence interval; NA: not available.

^a^ We calculated the 5-year average stillbirth rate from the rates reported in the annual sample registration system reports, 2016–2020.

^b^ Ladakh is included with Jammu and Kashmir.

Source: We obtained the sample registration system data from the reports available from the Office of the Registrar General & Census Commissioner India^27^ and the national family health survey data from the Demographic and Health Surveys website.^28^ The sample registration system does not provide confidence interval around the point estimates. The numbers of births, stillbirths and neonatal deaths were not reported in the sample registration system reports.

### Stillbirth documentation

According to the available details, the sample registration system process involves continuous enumeration of a given population to document births by monitoring the outcome of pregnancies in the population. The data are further checked on a half-yearly basis (Fig. 1). The enumerator manual lists the specific guidance and instructions to document pregnancy outcomes; the guidance relevant for stillbirth documentation is shown in Box 1. The duration of pregnancy is recorded in months, and stillbirth is defined based on 28 weeks of gestation. The guidance defines stillbirth as “the fetus does not breathe or show any other evidence of life”, but provides no clear indication of the meaning of “other evidence of life”. Miscarriage is documented under abortion (spontaneous abortion). Furthermore, the pattern of questions in the verbal autopsy tool indicates that the tool does not differentiate well between stillbirths and neonatal deaths occurring immediately after birth.^19^ There is a single question to establish the difference between a stillbirth and live birth, with all signs of life grouped together in one question which defines alive if the baby ever cried, moved or breathed. We could not identify any further guidance for identifying stillbirths in the verbal autopsy manual for supervisors. When we examined the Population Health Metrics Research Consortium and the WHO verbal autopsy tools,^19^ we found that they both follow the standard criteria for identification of stillbirths in verbal autopsy interviews. Each of the three signs of life (cry, move and breathe) are recorded separately to reduce misreporting between stillbirths and neonatal deaths immediately after birth. The babies who showed none of the three signs of life (did not cry *and* did not move *and* did not breathe) are then identified as stillbirths.

Box 1 **Instructions relevant to stillbirth documentation provided in the enumerator manual of the Indian sample registration system**

***Form no. 3: Pregnancy status of women***

**Column 8: Current pregnancy status**

Instructions: If pregnant write code 1, and if not, leave this column blank.

**Column 9: Duration of pregnancy in completed months**

Instructions: For women with code 1 in column 8 (currently pregnant), record here the duration of pregnancy in completed months, otherwise leave it blank.

**Column 11: Outcome of pregnancy for women with code 1 in column 8**

Instructions: If the outcome of pregnancy is livebirth, record 1 and if stillbirth, record 2. If pregnancy has been aborted, record 3.

***Form no. 4: Outcome of pregnancy recorded by enumerator***

**Column 12: Live birth, stillbirth or abortion (live birth code 1, stillbirth code 2, abortion code 3)**

Instructions: You should enquire about the outcome of pregnancy and write the code accordingly. For live birth write code 1, for stillbirth code 2 and for abortion code 3. The definitions of live birth, stillbirth and abortion are as follows:

- Live birth is a complete expulsion or extraction from its mother of a product of conception, irrespective of the duration of pregnancy, which after such separation, breathes or shows any other evidence of life, such as beating of the heart, pulsation of the umbilical cord or definite movement of voluntary muscles, whether or not the umbilical cord has been cut or the placenta is attached; each product of such a birth is considered as a live birth.
- Stillbirth is the fetal death before the complete expulsion or extraction from its mother of a product of conception, irrespective of the duration of pregnancy. The death is indicated by the fact that after such separation, the fetus does not breathe or show any other evidence of life. If such a product of conception has attained at least 28 weeks of gestation, it will be termed as a stillbirth.
- Abortion means termination of pregnancy. It is an immature outcome, which does not turn out to be a live one. There are two types of abortion. One is miscarriage and other is induced. The miscarriage is a natural abortion, which is beyond the control of the woman. However, the induced abortion is undergone intentionally. The natural abortion is known as spontaneous abortion, whereas the induced abortion is known as medical abortion. This type of abortion may be legal or therapeutic.

Source: We obtained the sample registration system statistical report 2020 from the Office of the Registrar General & Census Commissioner India.^27^

The national family health survey documents the full history of live births but only “the last pregnancy that resulted in either of the three adverse outcomes of interest (stillbirth, miscarriage and abortion)” (Fig. 2). In other words, only one adverse outcome of pregnancy is documented for a woman in the period of interest (5 years), irrespective of the number of adverse outcomes she may have had during that period. Therefore, the stillbirth rate from the survey refers to the stillbirths that were the last adverse pregnancy outcome in the time period of interest, and not all stillbirths during that period. The survey did not collect data on pregnancy, labour, delivery or postpartum care of the mother for pregnancies that resulted in a stillbirth.

## Discussion

Although it is widely known that the sample registration system underestimates stillbirths in India, we sought to examine the magnitude of under-reporting at the sub-national level in comparison with the national family health survey. We also looked at possible methodological issues in documenting stillbirths. The magnitude of the mismatch in stillbirth rates versus the similarity of neonatal mortality rates between the two data sources highlights the urgent need to improve the documentation of stillbirths in the sample registration system.

The process review we undertook is based on the documents available in the public domain for the sample registration system, and hence we could have missed small details in data collection that result in undercounting of stillbirths. The sample registration system uses prospective follow-up of outcomes of a given set of pregnancies by a trained enumerator, with data checked by a supervisor. In practice, this should leave little chance of error in the reporting of pregnancy outcomes either as live births or stillbirths, and of neonatal deaths. In contrast, the national family health survey requires respondents to retrospectively recall stillbirth as an adverse pregnancy outcome in the past 5 years, along with miscarriages and abortions. However, the prospective method of the sample registration system, which uses extensive checking, results in gross under-reporting of stillbirths but not of neonatal deaths as compared with the national family health survey. 

Assuming that the enumerator and supervisor for the sample registration system reach all or most pregnant women in the defined population to document pregnancy outcomes, we explored potential reasons why stillbirths are under-reported. First, if many stillbirths are misclassified as neonatal deaths, it should result in an over-estimation of neonatal mortality rate in the sample registration system as compared with the national family health survey, which is not the case. Second, if there is stigma in reporting of stillbirths in India, as in many other countries,^29^ then stigma would be applicable to the populations of both surveys, which does not seem to be the case, given the difference in stillbirth rates. Third, the under-reporting of stillbirths using the sample registration system may be due to misreporting of the gestation period, which is documented in months and not weeks. The national family health survey also documents the gestation period in months, but results in a higher stillbirth rate. Of concern, adjustment for gestational age in the national family health survey led to sizeable changes in the stillbirth rate in some states. This finding has serious implications for planning of stillbirth prevention interventions based on these data. 

Finally, in the sample registration system a pregnancy outcome is documented as livebirth, stillbirth and abortion, with miscarriage documented as spontaneous abortion. However, the women in the national family health survey were asked “Did that pregnancy end in a miscarriage, an abortion, or a stillbirth?”, clearly differentiating between (induced) abortion and miscarriage. Given that abortion in India is influenced by gender, cultural and legal issues, and that unsafe abortion is an important public health problem,^30^^–^^32^ it is likely that women under-report abortions in the sample registration system. Miscarriage too may be under-reported in the sample registration system because it is considered as abortion, and some of the under-reported miscarriages may be stillbirths, thereby leading to undercounting of stillbirths. We cannot comment on the abortion rate in the sample registration system as it is not available in the public domain. Comparison of the verbal autopsy tool with the two established international verbal autopsy tools showed that the documentation in the sample registration system does not allow for confirmation of a stillbirth as compared with neonatal death immediately after birth, as is done in the other two other tools. Concerns have been raised previously about the process and flow of data and the completeness of death data when using the sample registration system.^33^^,^^34^

To address the stillbirth undercount, we recommend that the sample registration system is adapted in the following ways: (i) to document induced abortions and miscarriages separately; (ii) to assess the quality and standardization of documenting gestation period for pregnancies; and (iii) to define stillbirths explicitly for the enumerators (for example, as babies who do not show any of the three signs of life). It may also be useful for the sample registration system to compare the abortion rate with that available from other studies, to understand the documentation issues. Given the extensive process of monitoring data quality in the sample registration system, our overarching recommendation is for supervisors to specifically review the number of stillbirths documented by enumerators in addition to the number of live births.

Although we estimated a higher stillbirth rate in the fifth round of the national family health survey than in the sample registration system for many states, stillbirths were still under-reported as the national family survey does not document full pregnancy history. Therefore, stillbirths are underrepresented in the national family health survey as compared with neonatal deaths. Limited population-based data on stillbirths are available from India to understand the extent of under-reporting of stillbirths in the national family health survey. The stillbirth rate for Bihar state in 2011–2014 from a population-based survey was estimated at 21.2 stillbirths per 1000 births (95% CI: 19.7–22.6), which was almost twice the direct rate in the fourth round of the national family health survey of 11.4 stillbirths per 1000 births.^35^ While full pregnancy history is still not documented in many demographic and health surveys globally, some countries do use the full pregnancy history.^36^^,^^37^ We have previously highlighted this issue for the national family health survey.^38^ Such an omission interferes with the availability of population-level data to inform action to end preventable stillbirths. We believe that if changes are made to the national family health survey to collect full pregnancy history in the next round of data collection,^37^ it will improve the robustness of stillbirth data for India.

Another important consideration for improving the stillbirth counts in India is to increase community awareness to improve the registration of stillbirths, which is mandatory under the Birth and Death Registration Act of India.^39^ Extrapolating for the estimated 340 622 stillbirths in India in 2019 as per the Inter-Agency Group report,^8^ only 159 645 (47%) of these estimated stillbirths were registered with the vital registration system.^11^

Our analysis highlights the invisibility of stillbirths due to the methods of data collection in the sample registration system used to track perinatal mortality in India. We also highlight the need to capture all stillbirths in India’s reproductive health and family planning surveys. For the India Newborn Action Plan to meet the 2030 target of a single-digit stillbirth rate, efforts are needed to improve the documentation of stillbirths in both surveys to track actions to end preventable stillbirths in India.
